# Drought stress and tree size determine stem CO_2_ efflux in a tropical forest

**DOI:** 10.1111/nph.15024

**Published:** 2018-02-03

**Authors:** Lucy Rowland, Antonio C. L. da Costa, Alex A. R. Oliveira, Rafael S. Oliveira, Paulo L. Bittencourt, Patricia B. Costa, Andre L. Giles, Azul I. Sosa, Ingrid Coughlin, John L. Godlee, Steel S. Vasconcelos, João A. S. Junior, Leandro V. Ferreira, Maurizio Mencuccini, Patrick Meir

**Affiliations:** ^1^ College of Life and Environmental Sciences University of Exeter Exeter EX4 4RJ UK; ^2^ Instituto de Geosciências Universidade Federal do Pará Belém PA 66075‐110 Brasil; ^3^ Museu Paraense Emílio Goeldi Belém PA 66040‐170 Brasil; ^4^ Instituto de Biologia UNICAMP Campinas SP 13083‐970 Brasil; ^5^ Departamento de Biologia FFCLRP Universidade de São Paulo Ribeirão Preto SP 14040‐900 Brasil; ^6^ School of GeoSciences University of Edinburgh Edinburgh EH9 3FF UK; ^7^ EMBRAPA Amazônia Oriental 14 Belém PA 66095‐903 Brasil; ^8^ CREAF Campus UAB Cerdanyola del Vallés 08193 Spain; ^9^ ICREA Barcelona 08010 Spain; ^10^ Research School of Biology Australian National University Canberra ACT 2601 Australia

**Keywords:** drought, growth respiration, maintenance respiration, stem CO_2_ efflux, tropical rainforests, woody tissue respiration

## Abstract

- CO_2_ efflux from stems (CO_2_stem_) accounts for a substantial fraction of tropical forest gross primary productivity, but the climate sensitivity of this flux remains poorly understood.
- We present a study of tropical forest CO_2_stem_ from 215 trees across wet and dry seasons, at the world's longest running tropical forest drought experiment site.
- We show a 27% increase in wet season CO_2_stem_ in the droughted forest relative to a control forest. This was driven by increasing CO_2_stem_ in trees 10–40 cm diameter. Furthermore, we show that drought increases the proportion of maintenance to growth respiration in trees > 20 cm diameter, including large increases in maintenance respiration in the largest droughted trees, > 40 cm diameter. However, we found no clear taxonomic influence on CO_2_stem_ and were unable to accurately predict how drought sensitivity altered ecosystem scale CO_2_stem_, due to substantial uncertainty introduced by contrasting methods previously employed to scale CO_2_stem_ fluxes.
- Our findings indicate that under future scenarios of elevated drought, increases in CO_2_stem_ may augment carbon losses, weakening or potentially reversing the tropical forest carbon sink. However, due to substantial uncertainties in scaling CO_2_stem_ fluxes, stand‐scale future estimates of changes in stem CO_2_ emissions remain highly uncertain.

## Introduction

Aboveground woody biomass is the largest store of carbon in tropical rainforests. The respiration from the stem and branch material within this woody pool has been estimated to account for 13–25% of total ecosystem respiration (Chambers *et al*., 2004; Cavaleri *et al*., 2006; Malhi *et al*., 2009) and 12–27% of gross primary productivity (Ryan *et al*., 1994; Chambers *et al*., 2004; Malhi *et al*., 2009; Doughty *et al*., 2015). However estimates of stem CO_2_ efflux (CO_2_stem_) remain highly uncertain in tropical forests, as only a handful of studies of CO_2_stem_ exist (Ryan *et al*., 1994; Meir & Grace, 2002; Malhi *et al*., 2009, 2013; Robertson *et al*., 2010; Angert *et al*., 2012; Katayama *et al*., 2014, 2016). Consequently, substantial inconsistency exists amongst studies concerning how CO_2_stem_ in tropical forests changes with tree height (Cavaleri *et al*., 2006; Katayama *et al*., 2014, 2016), with season (Cavaleri *et al*., 2006; Stahl *et al*., 2011) and across environmental gradients (Robertson *et al*., 2010), and how CO_2_stem_ scales with tree size and growth rate (Meir & Grace, 2002; Cavaleri *et al*., 2006; Katayama *et al*., 2016). Given the concern over tropical forests shifting from a global sink to a source of carbon as the climate changes (Lenton, 2011; Davidson *et al*., 2012; Brienen *et al*., 2015; Doughty *et al*., 2015), understanding how CO_2_stem_ varies with environmental change and how we calculate fluxes at ecosystem scales is becoming increasingly important.

The CO_2_ efflux from tree stems is likely to be mostly comprised of respiration derived from growth of new tissue (*R*_g_) and maintenance (*R*_m_) of existing tissues (McCree, 1970; Thornley, 1970; Ryan, 1990; Damesin *et al*., 2002; Meir & Grace, 2002). However CO_2_ efflux measured on trees may under‐ or overestimate stem respiration from the immediately underlying woody tissue due to other processes occurring within the trees, for example: high concentrations of CO_2_ in the soil, most likely from root respired CO_2_, being transported up to the site of measurement in sap (McCree, 1970; Levy *et al*., 1999; McGuire *et al*., 2007; Saveyn *et al*., 2008; Teskey *et al*., 2008; Aubrey & Teskey, 2009; Trumbore *et al*., 2013; Hillman & Angert, 2016); the transport of CO_2_ from below the point of measurement upwards in sap (Angert *et al*., 2012; Trumbore *et al*., 2013; Hilman & Angert, 2016); and nonphotosynthetic CO_2_ fixation by phosphoenolpyruvate carboxylase (PEPC) (Berveiller & Damesin, 2008). These processes can change over time with changes in sap pH, stem temperature, sap flow velocity or changes in gas diffusivity in the stem over time, which may arise from an increase in air‐filled spaces or even cracks in the bark (Cherubini *et al*., 1997; Levy *et al*., 1999; Sorz & Hietz, 2006; Teskey *et al*., 2008; Trumbore *et al*., 2013) Within tropical trees these processes have been relatively sparsely studied, in part due to the complexities of measuring such processes (Trumbore *et al*., 2013), particularly in what are often remote, challenging field locations. However, a new approach recently used in tropical forests combined oxygen consumption and CO_2_ efflux measurements to show that the apparent respiratory quotient of O_2_ to CO_2_ (ARQ) of tropical trees was less than the expected value of 1 (0.66 ± 0.18), suggesting that up to a third of CO_2_ was being transported away from the site of measurement causing underestimations of stem respiration (e.g. Angert *et al*., 2012). These results underline the notion that stem CO_2_ efflux measurements are likely to comprise signals from growth and maintenance respiration in combination with other stem processes, thus requiring caution when interpreting results.

Tropical forest growth and maintenance respiration (*R*_g_ and *R*_m_) components have generally been derived from linear regressions of CO_2_stem_ on growth rate (McCree, 1970; Thornley, 1970; Meir & Grace, 2002), with the intercept interpreted to give the maintenance respiration flux at zero growth rate. Due to the potential loss or gain of CO_2_ from other within‐stem processes, it is unlikely that these calculations give an entirely accurate representation of *R*_g_ or *R*_m_. If, however, we assume that CO_2_ is gained or lost equally from the CO_2_ produced by *R*_g_ or *R*_m_, such methods may still provide a good representation of the proportion of CO_2_ derived from growth and respiration, even if the quantitative values are not certain. Knowing these proportions is important because as trees experience climate stress it is likely that growth rates will decline (da Costa *et al*., 2010; Brienen *et al*., 2015; Korner, 2015), whilst simultaneous investment into maintaining existing tissues may rise (Metcalfe *et al*., 2010; Rowland *et al*., 2015b). Nonetheless, no studies in tropical forest have determined how growth and maintenance respiration change as mature tropical trees experience climate‐related stress, and how this is likely to influence stand‐scale CO_2_ efflux from woody tissue.

One of the key future climate changes which tropical forests are expected to experience in the coming decades is water stress caused by increased seasonal, interannual and decadal‐scale drought (Fu *et al*., 2013; Boisier *et al*., 2015; Duffy *et al*., 2015). Relative to photosynthetic fluxes, how respiration fluxes will respond to drought stress remains poorly constrained (Meir *et al*., 2008; Atkin & Macherel, 2009; Rowland *et al*., 2014). Limited data on temperate species suggest that stem CO_2_ efflux declines with water stress (Saveyn *et al*., 2007; Rodríguez‐Calcerrada *et al*., 2014). These studies agree with a number of studies on leaves, which find that leaf respiration is downregulated during short‐term drought stress, due to declining substrate availability (Ayub *et al*., 2011; Catoni & Gratani, 2014; Chastain *et al*., 2014; O'Brien *et al*., 2015). By contrast, some studies have shown increased leaf respiration with drought stress, particularly when drought occurs over extended periods (Miranda *et al*., 2005; Atkin & Macherel, 2009; Metcalfe *et al*., 2010; Rowland *et al*., 2015b; Varone & Gratani, 2015). Increased respiration during drought conditions may be expected if a greater amount of substrate is required for hydraulic repair and maintenance (Brodersen & McElrone, 2013), phloem transport regulation (Mencuccini & Hölttä, 2010) or oxidation of reactive oxygen species (Atkin & Macherel, 2009). Consequently, changes in respiration following drought are likely to be controlled by tree size and genera because trees of different sizes and genera have been shown to experience different hydraulic and metabolic costs as a consequence of drought stress (Rowland *et al*., 2015a,b), alongside having differing stem growth and maintenance costs.

However, a paucity of studies in tropical ecosystems, and globally, means that our current understanding of how CO_2_stem_, one of the largest components of autotrophic respiration, will respond to future increases in water stress still remains highly uncertain. This uncertainty is amplified by the existence of various methods for scaling these fluxes to the ecosystem, including according to total stem area or sapwood volume (e.g. Levy & Jarvis 1998; Cavaleri *et al*., 2006; Katayama *et al*., 2014), which result in large differences in ecosystem‐scale estimates of stem CO_2_ release. In the present study, we report the results from a study of CO_2_stem_ on 215 trees in dry and wet seasons, in a forest that has experienced 15 yr of experimental drought and in adjacent corresponding control forest. Using these data we test the following hypotheses: drought causes an increase in CO_2_stem_, due to increasing maintenance costs associated with low moisture availability; CO_2_stem_ will be significantly different among genera, as metabolic processes and responses to drought are taxonomically conserved; long‐term drought increases the proportion of maintenance to growth respiration, as a consequence of increasing maintenance costs and reducing growth; and the effect of long‐term drought on stand‐scale estimates of CO_2_stem_ changes according to whether CO_2_stem_ rates are scaled using estimates of total stem area or of sapwood volume.

## Materials and Methods

### Site

The study was performed at a through‐fall exclusion (TFE) experiment in the Caxiuanã National Forest reserve in eastern Amazonia (1°43′ S. 51°27′ W). The site is 15 m above sea level, located within *terra firme* forest on yellow oxisol soils (Ruivo & Cunha, 2003). It experiences a mean annual rainfall of 2000–2500 mm and a pronounced dry season in the later 6 months of the year.

The experiment comprised two 1‐ha plots, a control plot with no drought infrastructure and a TFE where plastic panels and guttering at 1–2 m in height are used to exclude 50% of the canopy through‐fall from reaching the forest floor (da Costa *et al*., 2010). Both plots were trenched to 1–2 m to prevent lateral flow of water in the soil. To maintain biogeochemical inputs into the soil, leaf litter on the TFE panels is relocated to the forest floor every few days. The TFE treatment has been maintained since January 2002, and therefore before this study all trees on the TFE had experienced 15–16 yr of a 50% reduction in canopy through‐fall. Further details on the experiment can be found in da Costa *et al*. (2010) and Meir *et al*. (2015).

### Sample selection

Measurements were performed on 215 trees in total, 105 from the control plot and 110 from the TFE during October 2016 (mid dry season) and April 2017 (mid wet season). First, we selected trees from 12 of the most common genera found on both the control plot and the TFE (*Aspidosperma*,* Eschweilera*,* Inga*,* Licania*,* Micropholis*,* Minqurtia*,* Pouteria*,* Protium*,* Swartzia*,* Syzygiopsis*,* Virola* and *Vouacapoua*), totalling 87 and 77 trees on the control and the TFE plots, respectively. The remainder of the trees – 18 on the control plot and 33 on the TFE plot – comprised trees with a diameter at breast height (dbh) > 30 cm on the TFE and > 40 cm on the control, measured to ensure more equal division of trees amongst size classes. From October 2013 to January 2016, seven measurement campaigns were also carried out on 16–18 trees on the control and 19–20 trees on the TFE, of the genera (*Eschweilera*,* Licania*,* Manilkara*,* Pouteria*,* Protium* and *Swartzia*) previously sampled for photosynthesis measurements by Rowland *et al*. (2015b). A list of all of the species samples in each measurement campaign from 2013 to 2017 is presented in Supporting Information Table S1.

### CO_2_stem_ measurements

CO_2_stem_ was measured using a transparent acrylic chamber, temporarily sealed onto the stem surface using a closed cell non‐CO_2_ adsorbent foam gasket and two ratcheting straps. The chamber was sealed to the stem at a constant gasket thickness and had a volume of 213 cm^3^ (including tubing and foam) and a surface area of 75 cm^2^ of the bark surface. The chamber size and construction were similar to those used for other measurements of CO_2_ efflux in tropical forests (Stahl *et al*., 2011; Rowland *et al*., 2013). The chamber was connected to an infrared gas analyser (EGM4, EGM5; PPSystems, Hitchen, UK) for 220 s and was used to detect an increase in CO_2_ concentration inside the chamber. Following Rayment & Jarvis (2001), to promote air mixing in the chamber without creating vortex effects from the operation of a fan, the chamber also contained a 15‐cm length of tube perforated with 0.5 mm diameter holes, connected to the inlet. During each measurement we tested for leaks by exposing the edges of the chamber to very high CO_2_ concentrations. If any increase in CO_2_ concentration inside the chamber was detected, the measurement was aborted. Wood temperature (*T*_w_) was measured using a type T thermocouple placed into the bark, or where this was not possible, on the bark surface. All measurements were made between 08:00 h and 14:00 h.

Measurements of the increase in CO_2_ concentration between 120 and 220 s were used for analysis, leaving 2 min for the chamber to stabilize post‐installation. The slope of the linear regression between time and CO_2_ was extracted to calculate CO_2_stem_ (stem CO_2_ efflux, μmol m^−2^ s^−1^) using Eqn (Eqn 1)

(Eqn 1) $${\text{CO}}_{2\_\mathrm{stem}}=\frac{\Delta {\text{CO}}_{2}}{\Delta t}\times \frac{V_{c}}{S_{c}}\times a\times \frac{273.15}{273.15+T_{w}},$$

(ΔCO_2_/Δ_*t*_, slope of the CO_2_–time relationship; *V*_c_ volume (cm^3^) and *S*_c_ the surface area (cm^2^) of the chamber; *a*, volume of a mole of CO_2_ (mol cm^3^); *T*_w_, measured wood temperature (°C)). Linear slope values with a correlation coefficient < 0.98 were discarded from the analysis and the data were temperature‐corrected to 25°C using a *Q*_10_ of 2.0 (Cavaleri *et al*., 2006). After excluding measurements with leaks or with a correlation coefficient < 0.98, 97 measurements were included on the control plot and 108 on the TFE plot from the dry season; and 97 from the control and 99 from the TFE plots, respectively, were included from the wet season.

### Diurnal tests

In order to test for daytime increases in stand‐scale CO_2_stem_ (S_CO_2_stem_), which could result in biases according to the time CO_2_stem_ was measured or indicate other forms of CO_2_ transport or consumption (Teskey *et al*., 2008; Angert *et al*., 2012), we measured CO_2_stem_ every 15 s for 24 h on 20 trees from the control and the TFE in October 2013, using an open path respiration system similar to that used elsewhere (Rayment & Jarvis, 2001; Meir & Grace, 2002) and a CIRAS 1 IRGA (PPSystems); for further details see Methods S1). We found very limited diurnal variation in CO_2_stem_, indicating limited bias concerning the time of day the measurements were taken (see Fig. S1 and Methods S1).

### Growth data

Quarterly mean tree‐level stem diameter increment per plot from 2010 to 2015 were taken from dendrometer measurements presented in Rowland *et al*. (2015a), and updated to the end of 2016 following the same methodology and converted to units of cm d^−1^. A long‐term annual increment then was calculated for each tree based on the 2010–2016 dataset. This interval (2010–2016) was chosen as it represented the period after which the growth rates of the small and medium trees on the TFE (10–40 cm dbh) had re‐stabilized following increased growth rates in response to elevated light intensities (see Rowland *et al*., 2015a for further details). We note that accurate growth measurements were not available for some of the larger trees in this study, as it was not feasible to monitor these trees on a three‐monthly basis due to their size or because a dendrometer could not be accurately fitted on the tree due to substantial trunk‐shape irregularities.

### Scaling

Scaling was performed using three methods, which are described in detail in Methods S1. The three methods were used to assess the effect of different scaling assumptions on S_CO_2_stem_ estimates. Method one (M1) involved scaling according to total stem surface area. Method two (M2) used estimated total sapwood volume as the scalar. Initially we estimated total sapwood volume to be 34% of total tree volume (an estimate of the sapwood area: basal area ratio at 1.3 m above ground level; see Methods S1 and Fig. S2) and then, given that 34% is likely to underestimate the greater percentage sapwood area in smaller diameter branches, we assessed how this calculation changed using an estimate of 50% and 80% sapwood volume. We assumed constant live‐cell fraction in all sapwood volume estimates. Method 3 (M3) involved a combination of the two scaling methods above. Following Cavaleri *et al*. (2006), but taking a total sapwood volume approach, we assumed that for any part of the canopy < 10 cm in diameter CO_2_stem_ scaled with total stem surface area, and for sections > 10 cm CO_2_stem_ was scaled with total sapwood volume. For all methods trees within 10 m of the edge of the plots were excluded from our calculations to eliminate possible long‐term effects of the trenching on the community structure and tree physiology (da Costa *et al.,* 2010). Wet and dry season S*_*CO_2_stem_ estimates from all scaling methods were averaged and converted to units of Mg C ha^−1^ yr^−1^.

### Analysis

All statistical analyses were performed in the statistical package R (v.3.4.0; R Core Team, 2017) and all errors are shown as standard errors on the mean, but do not account for the sampling error of the calibration of the gas analyser (< 1% in EGM). Following Damesin *et al*. (2002) and Meir & Grace (2002), we calculated averaged plot‐level maintenance respiration as the intercept of the relationship between growth and CO_2_stem_, but using a bootstrapping technique, to avoid assumptions about normality of distribution and to facilitate the calculation of errors. First we randomly sampled our study trees, with replacement, to create 1000 samples of the trees which had growth and CO_2_ efflux data on each plot (75 control, 87 TFE). Following this, we calculated 1000 estimates of: mean total CO_2_stem_ per tree, for each plot; the *y*‐intercept of the woody increment– CO_2_stem_ relationship (*R*_m_); and *R*_g,_ calculated as mean CO_2_stem_ minus *R*_m_. Mean and SE values of CO_2_stem_, *R*_m_ and *R*_g_ per tree for each plot were then calculated from the mean and SD of the bootstrapped samples. Data comparisons of the proportions of *R*_m_ and *R*_g_ between plots, seasons and tree size classes (small: 10–20 cm dbh, medium: 20–40 cm dbh and large: > 40 cm dbh) were then made. Given that the bootstrapping created a normal distribution, statistical comparisons of CO_2_stem_, *R*_m_ and *R*_g_ were made using a parametric paired *t*‐test and only percentage values of *R*_m_ and *R*_g_ are presented, acknowledging that absolute values are uncertain because of uncertainties in estimating woody respiration from CO_2_stem_ (Teskey *et al*., 2008; Trumbore *et al*., 2013).

Analysis of whether CO_2_stem_ scales with surface area or sapwood volume was performed following Levy & Jarvis (1998). Log‐transformed linear relationships were created for CO_2_stem_ (μmol m^−2^ s^−1^) against dbh and CO_2_stem_ (μmol m^−3^ s^−1^) against 1/dbh. A significant relationship between area‐based CO_2_stem_ and dbh indicates that a scaling relationship with volume exists, and a significant relationship between volume‐based CO_2_stem_ and 1/dbh indicates that a scaling relationship with area exists (Levy & Jarvis, 1998). Consequently the slopes of these relationships indicate the proportional scaling with volume or area (respectively) as, for example, a slope of 1 between dbh and CO_2_stem_ (μmol m^−2^ s^−1^) would indicate perfect volume scaling, whilst a slope of 0 would indicate perfect area scaling (see Levy & Jarvis, 1998).

## Results

### Drought response of CO_2_stem_

**Figure 1:**
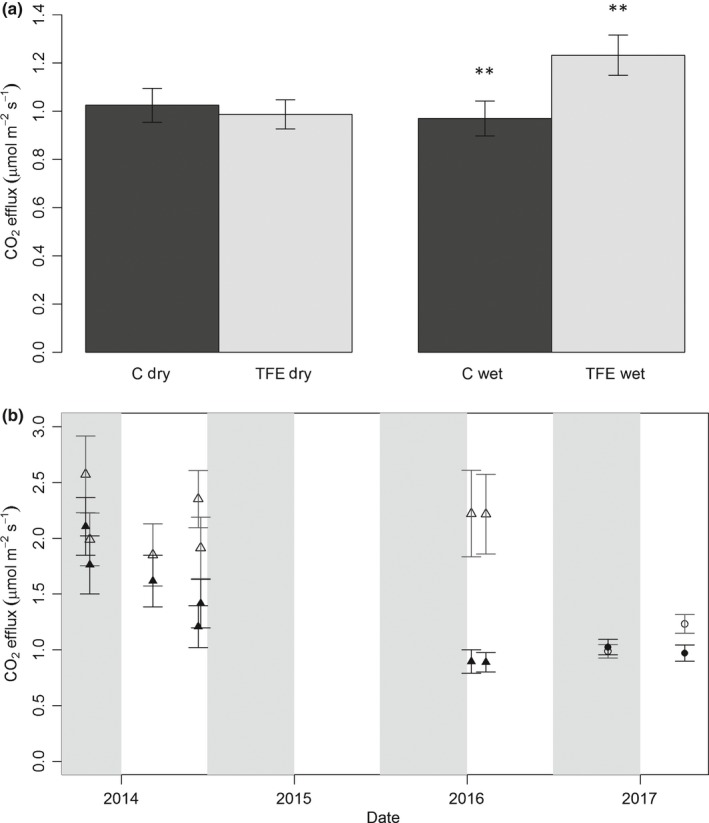
(a) Stem CO_2_ efflux (μmol m^−2^ s^−1^) in mid dry season (October 2016) and mid wet season (April 2017) on the control (C, black) and through‐fall exclusion (TFE) plot (grey). Asterisks indicate significant increase at *P *<* *0.01 between columns. (b) Stem CO_2_ efflux (μmol m^−2^ s^−1^) on a time series of data from the control (closed symbols) and the TFE (open symbols) plot. Grey shaded areas show dry season months (July‐December), triangles indicate measurements taken with *n *=* *21 individuals per plot, and circles indicate measurements taken with *n *=* *105 on the control and *n *=* *110 on the TFE (see the Materials and Methods section and Supporting Information Table S1). Error bars indicate ± SE.

The CO_2_stem_ rates of trees on the control plot averaged 1.00 ± 0.10 μmol m^−2^ s^−1^ across both seasons, showing significantly higher CO_2_stem_ values in the dry season (dry = 1.01 ± 0.08 μmol m^−2^ s^−1^, wet = 0.87 ± 0.07 μmol m^−2^ s^−1^; *P *<* *0.01; Fig. 1a). By contrast, on the TFE plot there was a significant increase in CO_2_stem_ during the wet season relative to the control plot and the dry season (*P *<* *0.01, dry = 0.99 ± 0.06 μmol m^−2^ s^−1^, wet = 1.23 ± 0.08 μmol m^−2^ s^−1^). This represented a 27% increase in CO_2_stem_ on the TFE during the wet season relative to the control plot, a seasonal increase on the TFE plot itself of 24% relative to the dry season, and therefore an overall 11% increase in the mean wet and dry season CO_2_stem_ on the TFE relative to the control plot (Fig. 1a). Data from a previous analysis of 21 trees (see the Materials and Methods section, Methods S1 and Table S1) per plot measured six times between October 2013 and February 2016, also confirmed that the TFE tended to have consistently higher fluxes than the control plot during the wet season and more equal fluxes during the dry season (Fig. 1b). However, we note that the magnitude and plot differences in these latter flux values are likely to be less reliable due to a lower sample size.

**Figure 2:**
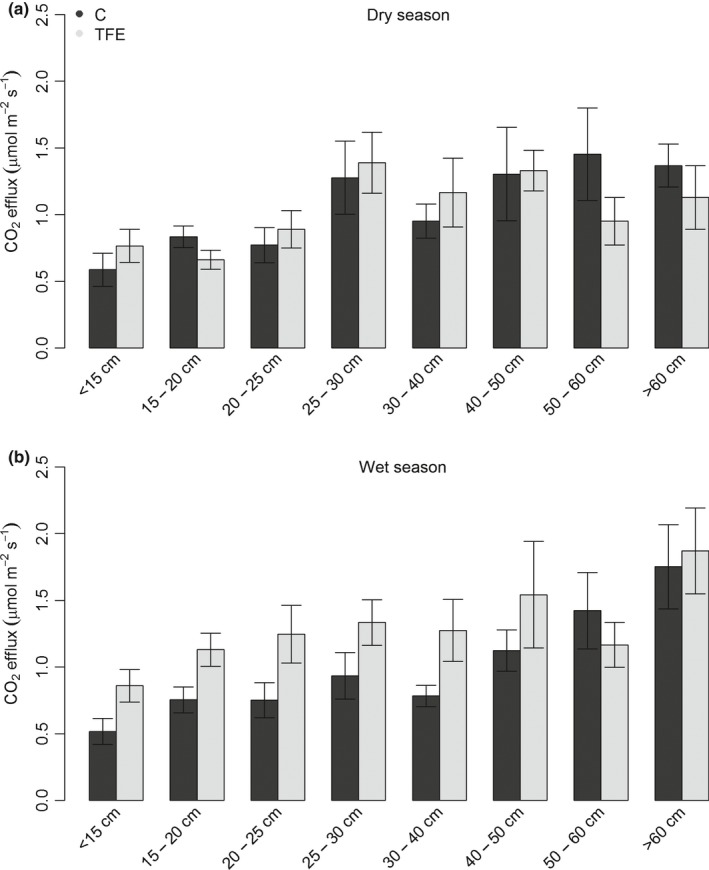
Mean stem CO_2_ efflux (μmol m^−2^ s^−1^) in (a) mid dry season (October 2016) and (b) mid wet season (April 2017) on the control (C, black) and through‐fall exclusion (TFE) plot (grey) for trees divided into diameter at breast height (1.3 m; dbh) size classes of < 15, 15–20, 20–25, 25–30, 30–40, 40–50, 50–60 and > 60 cm. Error bars show ± SE.

The increase in CO_2_stem_ on the TFE was controlled predominantly by significant increases in CO_2_stem_ from trees smaller than 40 cm dbh, which occurred in the wet, but not the dry season (Fig. 2). Interestingly, CO_2_stem_ increased with tree size on both plots, and this increase was more pronounced in the wet season and on the control plot (Fig. 2), where CO_2_stem_ of the largest tree size class (> 60 cm dbh) was 3.4‐fold greater than that for the smallest (< 15 cm dbh; Fig. 2b). On the TFE, due to the elevated CO_2_stem_ in the smallest trees, this increase in CO_2_stem_ from the smallest to the largest trees was reduced to 2.6‐fold.

### Taxonomic patterns in CO_2_stem_

**Figure 3:**
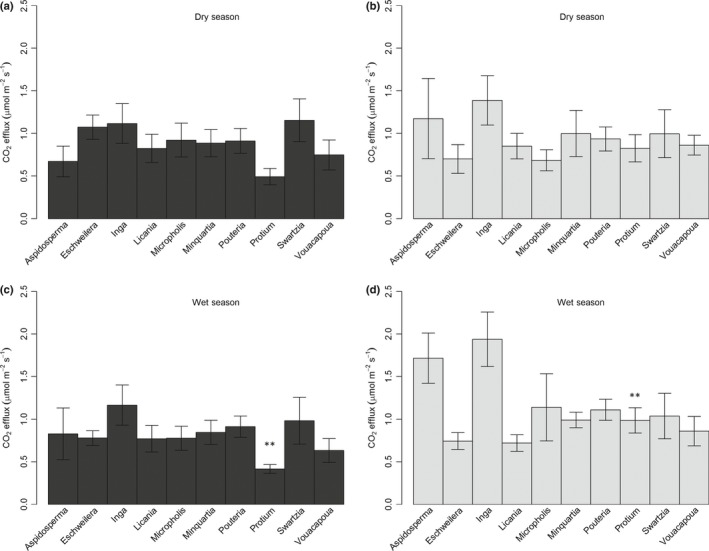
Mean stem CO_2_ efflux (μmol m^−2^ s^−1^) in (a, b) mid dry season (October 2016) and (c, d) mid wet season (April 2017) on (a, c) the control (C, black) and (b, d) through‐fall exclusion (TFE) plot (grey) for trees divided into genus groups, with greater than two individuals per group (see Supporting Information Table S1). Error bars show ± SE. Matching symbols indicate that columns are different at *P *<* *0.05.

Strong changes in CO_2_stem_ with tree size resulted in high variation in CO_2_stem_ within each genus (Fig. 3). Consequently, no significant differences were found among genera on each plot in dry season (Fig. 3). *Protium* was found to have significantly elevated CO_2_stem_ on the TFE, relative to the control during the wet season, although it did not demonstrate a significant increase from dry to wet season on the TFE (Fig. 3b,d). It is also noteworthy that, excluding *Protium* on the control and *Aspidosperma* and *Inga* on the TFE, the mean values per genus are largely similar within and between plots, as well as between seasons. These results suggest that CO_2_stem_ drought responses are not strongly taxonomically conserved.

### Growth and maintenance respiration

**Figure 4:**
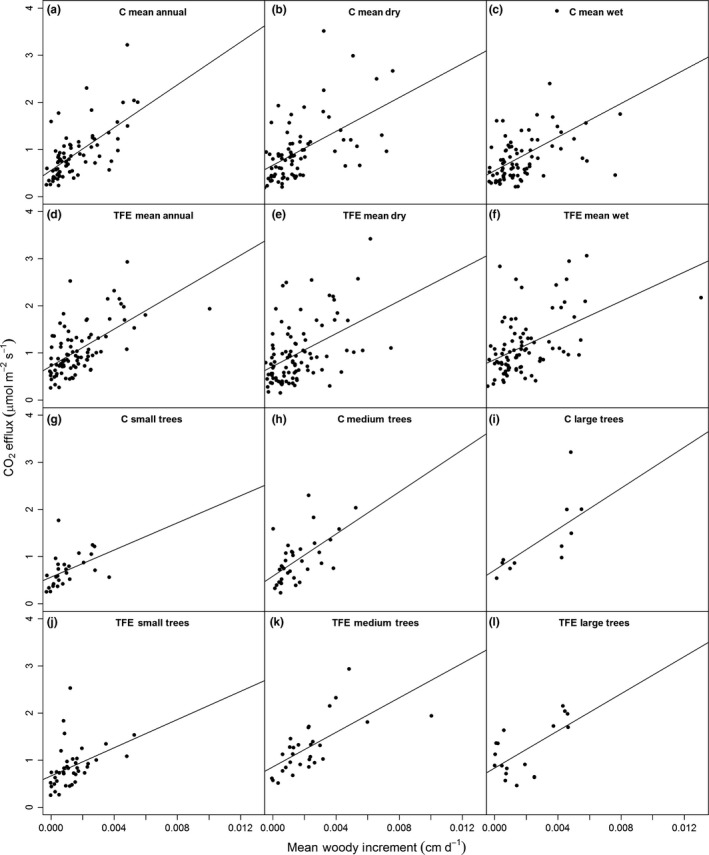
Relationships between mean stem CO_2_ efflux (μmol m^−2^ s^−1^) and the 2010–2015 mean annual woody increment (cm d^−1^) for the control (C) and through‐fall exclusion (TFE) plots for (a, d) mean annual stem CO_2_ efflux, (b, e) dry season CO_2_ efflux, (c, f) wet season stem CO_2_ efflux, and mean annual CO_2_ efflux split into (g, j) small (10–20 cm), (h, k) medium (20–40 cm) and (i, l) large trees (> 40 cm). Linear fit lines indicate significant (*P *<* *0.05) linear relationships. Correlation coefficients, *P*‐values and intercepts are shown in Table 1.

**Figure 5:**
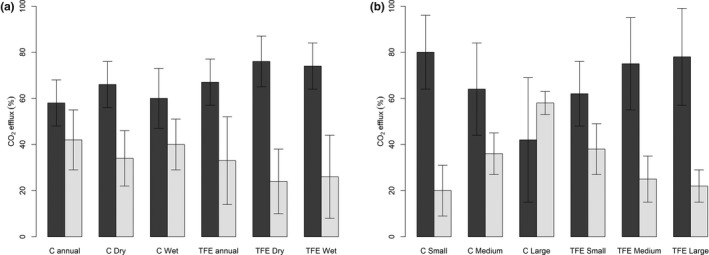
Estimated percentage of maintenance respiration (black) and growth respiration (grey) for the control plot (C) and through‐fall exclusion (TFE) plot, divided by (a) plot and season and (b) by tree size, averaging respiration across seasons. Error bars show ± SE.

Relationships between CO_2_stem_ and mean woody increment for 2010–2016 were performed on a *per* tree basis separately for mean annual total (wet and dry season), wet season and dry season CO_2_stem_, and for mean annual CO_2_stem_ divided into small, medium and large size classes. On both plots CO_2_stem_ by season or size class always had a positive and significant (at least *P *<* *0.01) relationship with mean wood increment (Fig. 4; Table 1). These relationships had a larger *r* ^2^ values on the control plot (e.g. *r* ^2^ control plot annual mean = 0.61, TFE plot annual mean = 0.37; Table 1); however, there were also consistently greater *r* ^2^ values in larger trees compared to small trees on both plots (Fig. 4; Table 1). When the percentage *R*_m_ and *R*_g_ values were estimated from these relationships, we find that on an annual basis the CO_2_ efflux associated with *R*_m_ accounts for 58 ± 10% and 67 ± 10% of total respiration on the control and TFE plot, respectively (Table 1; Fig. 5a). Furthermore, we find limited seasonal change in these values when averaging across trees of all size classes (Fig. 5a; Table 1). When trees were divided into size classes there were, however, strong shifts in the percentage division of *R*_m_ and *R*_g_. On the control plot in the small trees 80 ± 10% of the respiration was *R*_m_, and this declined to 60 ± 22% and 43 ± 27% in the medium and large trees, respectively (Fig. 5b; Table 1). By contrast, on the TFE the small trees had a lower percentage *R*_m_, 62 ± 14%, and this increased in the medium and large trees to 75 ± 20% and 78 ± 21%, respectively (Fig. 5b; Table 1). This suggests that *R*_m_ increases substantially in larger trees as a consequence of drought.

**Table 1:** Intercept (Int.), correlation coefficient (*r* ^2^) and *P*‐values (*P*), mean total stem CO_2_ efflux (CO_2_stem_; standard error given as CO_2_stem_se_) and the percentage (%) of total CO_2_ efflux associated with *R*_m_ and *R*_g_ for the panels in Fig. 5 (pan.) representing CO_2_stem_ values on the control (C) and through‐fall exclusion (TFE) averaged annually, for the wet and dry seasons, and average annual values separated by tree size (small, 10–20 cm diameter at breast height (dbh); medium, 20–40 cm dbh; large > 40 cm dbh)

Panel	Variable	*r* ^2^	*P*	Int.	CO_2_stem_	CO_2_stem_se_	% *R*_m_	% *R*_g_
(a)	C annual	0.61	0.00	0.55	0.95	0.07	58	42
(b)	C dry	0.44	0.00	0.68	1.02	0.08	66	34
(c)	C wet	0.44	0.00	0.53	0.89	0.08	60	40
(d)	TFE annual	0.37	0.00	0.72	1.07	0.06	67	33
(e)	TFE dry	0.17	0.00	0.75	0.99	0.07	76	24
(f)	TFE wet	0.25	0.00	0.85	1.15	0.07	74	26
(g)	C small	0.19	0.01	0.56	0.70	0.06	80	20
(h)	C medium	0.41	0.00	0.58	0.98	0.11	60	40
(i)	C large	0.73	0.00	0.68	1.59	0.31	43	57
(j)	TFE small	0.14	0.01	0.67	1.07	0.10	62	38
(k)	TFE medium	0.46	0.00	0.80	1.07	0.10	75	25
(l)	TFE large	0.36	0.01	0.83	1.07	0.16	78	22

### Scaling CO_2_stem_

**Figure 6:**
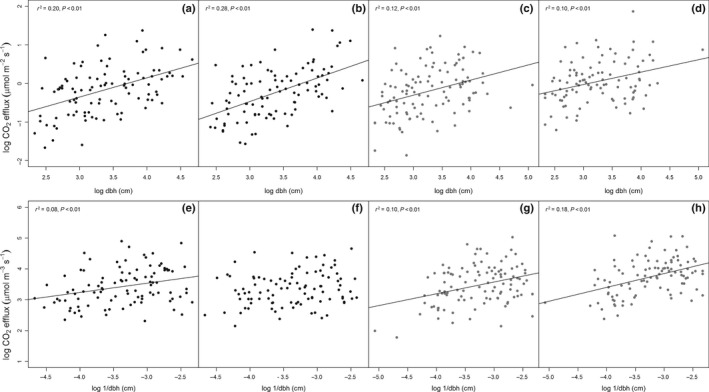
Relationships between log stem CO_2_ efflux by area (μmol m^−2^ s^−1^) and log diameter at breast height (dbh, cm) for the control (black) and through‐fall exclusion (TFE) (grey) plot in (a, c) dry and (b, d) wet. Relationships between log stem CO_2_ efflux by volume (μmol m^−3^ s^−1^) and log_1_/diameter are also shown for the control and TFE plot in (e, g) dry and (f, h) wet season. Linear regression fits, *r* ^2^ and *P‐*values are shown for significant (*P *<* *0.05) relationships.

On the control plot in both the wet and dry season data, there was a stronger correlation between log‐transformed CO_2_stem_ on an area basis (*r* ^2^ = 0.20–0.28) and dbh, than on a volume basis and 1/dbh (*r* ^2^ = 0.08; Fig. 6a,b,e,f). On the TFE plot, the relationships with dbh and 1/dbh were generally weaker than on the control plot (*r* ^2^ = 0.10–0.18; Fig. 6). However, on both the control and the TFE such low *r* ^2^ values created substantial uncertainty concerning whether area or volume is a better scalar; for example, the slope values for CO_2_stem_ by area against dbh and CO_2_stem_ by volume against 1/dbh in the dry season indicated a range of the percentage of the data which scaled with area from 50 ± 10% to 70 ± 10% on the control plot and of 39 ± 10% to 62 ± 10% on the TFE.

When we scaled up the CO_2_stem_ values to S*_*CO_2_stem_ for each plot, the various estimates for the stand‐scale flux of the control ranged by 4.7 Mg C ha^−1^ yr^−1^ and those of the TFE plot by 5.1 Mg C ha^−1^ yr^−1^ (Table 2). Furthermore, the percentage reduction in the S*_*CO_2_stem_ on the TFE relative to the control ranged from 0.7–22.9%, depending on the method of scaling (Table 2). The highest estimates of S*_*CO_2_stem_ came from using surface area as the scalar; however, these values were similar to the scaling outcome using the method of assuming volume as the scalar for wood < 10 cm diameter and area as the scalar for bole diameters > 10 cm. The area and the area–volume scaling methods both produced very small percentage differences between the control and the TFE S*_*CO_2_stem_. By contrast, scaling by sapwood volume alone produced substantially larger differences between the plots (in both absolute and relative terms), which were well‐conserved across the range of percentage sapwood volume used (34–80%). Scaling by sapwood volume produced far lower *S_*CO_2_stem_ values, which were also highly sensitive to the percentage value of sapwood volume used (Table 2).

**Table 2:** StemCO_2_ efflux (CO_2_stem_) values scaled to plot level (Mg C ha^−1^ yr^−1^) for the control and the through‐fall exclusion (TFE) plots, calculated according to: surface area scaling; volume scaling assuming 34%, 50% and 80% of the volume is sapwood (SW); scaling assuming CO_2_stem_ scales with volume for tree boles < 10 cm and with area for all woody sections > 10 cm diameter at breast height (dbh)

	Control	TFE	Change (%)
Surface area	7.07 ± 0.72	6.94 ± 0.63	1.8
Volume, 80% SW	5.53 ± 0.56	4.26 ± 0.39	22.9
Volume, 50% SW	3.46 ± 0.35	2.67 ± 0.24	22.8
Volume, 34% SW	2.40 ± 0.24	1.86 ± 0.17	22.5
Volume bole > 10 cm, area < 10 cm	6.81 ± 0.69	6.76 ± 0.61	0.7

Error term shows the ± SE propagated from the error on the measured CO_2_ efflux values only. The final column demonstrates the percentage change of the TFE relative to the control. The frequency distribution of trees across size categories for each plot can be seen in Supporting Information Table S2.

## Discussion

Using the world's longest‐running drought experiment in tropical forest and measurements of CO_2_ efflux from 215 stems in the wet and dry seasons, we demonstrated that the efflux of CO_2_ from stems (CO_2_stem_) increased by 27% on drought‐treated TFE (through‐fall exclusion) trees relative to control trees in the wet season. The increases in CO_2_stem_ were caused by large increases, of up to 40%, in the efflux rate of CO_2_ released from trees < 40 cm diameter at breast height (dbh) in the wet season, increases which were absent in the dry season. Furthermore, we found that there was a substantial increase in the percentage of total respiration that is associated with respiration resulting from maintenance (*R*_m_) on the TFE relative to the control, driven by reduced efflux associated with respiration resulting from growth (*R*_g_) and increased efflux associated with *R*_m_ in the medium and large trees. Finally we show that the stand‐scale CO_2_stem_ (S*_*CO_2_stem_) estimates, as well as the differences in S*_*CO_2_stem_ between plots are highly sensitive to the scaling method used, with absolute values varying by > 300% within plots and the percentage change between the plots varying by up to 22%.

Following 15 yr of rainfall exclusion, wet season CO_2_stem_ rates on the TFE plot were 27% higher (Figs 1, 3). This result contrasts with findings in temperate forests, where CO_2_stem_ declined, but with short‐term water stress (Saveyn *et al*., 2007; Rodríguez‐Calcerrada *et al*., 2014). However, our result is consistent with several reports elsewhere of drought‐related increases in respiration (Miranda *et al*., 2005; Varone & Gratani, 2015) and corroborates previous results from this site which showed substantial increases in leaf dark respiration on the TFE plot following extended periods of reduced soil moisture availability (Metcalfe *et al*., 2010; Rowland *et al*., 2015b), and evidence of coupled increases in root respiration (Metcalfe *et al*., 2007; Meir *et al*., 2008). Given that the elevated CO_2_stem_ occurs only in the wet season, we speculate that this could be caused by increased growth rates in the small and medium trees found to occur on the TFE (Rowland *et al*., 2015a) or potentially because the xylem tissue is undergoing hydraulic recovery (Brodersen & McElrone, 2013), following high hydraulic stress which is likely to occur during periods of extreme vapour pressure deficit (VPD) and low rainfall during the dry season on the TFE (Rowland *et al*., 2015a). This hypothesis is supported further by the significant increase in percentage of *R*_m_ on the TFE relative to the control during the wet season (Fig. 1a; Table 1), suggesting that the cost of maintaining existing tissues may be substantially higher on the TFE plot, especially in the largest trees.

Previously, maintenance respiration was estimated to comprise *c*. 80% of total respiration in mature trees in closed tropical rainforests (Ryan *et al*., 1994; Meir & Grace, 2002). Our analysis indicates that the division of CO_2_stem_ associated with *R*_g_ and *R*_m_ varies substantially by tree size class and with drought in tropical forest. On the control plot there was a strong trend toward decreases in percentage *R*_m_ with increasing tree size and increasing percentage *R*_g_ (Fig. 5b). This strong percentage decline in *R*_m_ with tree size was absent from the TFE plot trees, where percentage *R*_g_ declined with tree size (Table 1; Fig. 5b). Instead, on the TFE we observed a substantial increase in *R*_m_ in the largest trees relative to the control plot (Fig. 5b). As the largest trees are mostly likely to suffer damage, particular hydraulic damage, following drought stress (Bennett *et al*., 2015; McDowell & Allen, 2015; Rowland *et al*., 2015a), these results may suggest that these trees are unable to invest as much carbohydrate resource into *R*_g_. This may be driven by elevated maintenance costs associated with repairing drought‐damaged cells, removing reactive oxygen species, elevated phloem transport regulation or repair and/or replacement of hydraulically damaged xylem tissue. However, we note that the errors on our estimates of maintenance respiration are large for certain tree size classes (Table 1), due to smaller proportions of variance in CO_2_stem_ being explained by growth in some size classes than others. This may suggest that other unmeasured interaction variables are necessary to quantify the proportions of growth and maintenance respiration with greater accuracy.

In our analysis, we find no clear evidence of whether scaling by surface area or sapwood volume is more appropriate (Fig. 6). However we note that having used a relationship to estimate sapwood volume, we have estimates of sapwood volume, rather than a direct measurement and CO_2_stem_ may be more prone to error when calculated on a sapwood volume basis, than when calculated on a surface area to CO_2_stem_. Consequently we tested a variety of scaling methods to estimate our fluxes at the plot level. Competitive release of smaller trees on the TFE plot following a 40% loss of biomass from the mortality of the largest trees (da Costa *et al*., 2010; Rowland *et al*., 2015a) enhanced the growth and recruitment of the smallest size‐class trees, which also have the largest surface area to volume ratio. This shift in size distribution caused the TFE plot to have S*_*CO_2_stem_ that was almost equal to the S*_*CO_2_stem_ for the control plot when surface area, or mostly surface area‐based scaling was used, but substantially lower S*_*CO_2_stem_ when volume was used as the scalar.

Scaling by area is the most common form of scaling of CO_2_stem_ to the canopy (Chambers *et al*., 2004; Malhi *et al*., 2013). Given the radial live‐cell distribution in woody tissue it is unlikely, particularly in large diameter woody sections, that CO_2_stem_ scales directly with area, because CO_2_ production occurs in the living sapwood and phloem tissue (Fig. 5; Meir & Grace, 2002; Cavaleri *et al*., 2006; Levy & Jarvis, 1998). Scaling by sapwood volume does, however, introduce very large uncertainties into S*_*CO_2_stem_ estimates (Table 2), because the proportion of tree volume that is sapwood remains uncertain, as does the fraction of sapwood cells that are metabolically active. How sapwood volume scales with diameter within trees and between species in tropical forests is very sparsely studied (Meir *et al*., 2017), with no current estimates on how to calculate the sapwood volume of a tree (including the canopy), or its variation among species. In addition, the allometric scaling equations used for calculating tree volume and surface area (Methods S1) are also likely to introduce large errors into S*_*CO_2_stem_ estimates, the magnitudes of which are hard to estimate. Biomass studies have shown this may be particularly true for the largest trees (Calders *et al*., 2015), and this may suggest that greater unknown error exists in the S*_*CO_2_stem_ value for the control plot, where there are more large trees.

Throughout this study we present all absolute measured values as CO_2_stem_ while acknowledging that there are likely to be many other processes occurring within the stem, which may result in raw chamber‐based measurements of CO_2_ efflux from the stem, leading to over‐ or underestimates of the actually woody stem respiration underlying the measurement chamber (McCree, 1970; Levy *et al*., 1999, McGuire *et al*., 2007; Berveiller & Damesin, 2008; Saveyn *et al*., 2008; Teskey *et al*., 2008; Aubrey & Teskey, 2009; Angert *et al*., 2012; Trumbore *et al*., 2013; Hilman & Angert, 2016). However, we do note that we found limited diurnal changes in CO_2_stem_ (Fig. S1), suggesting, as found elsewhere (Ubierna *et al*., 2009; Stahl *et al*., 2011), that the upward transport of ‘excess’ CO_2_ from the soil or roots or the upward transport of CO_2_ from the point of measurement may be limited in this forest, or compensated for by other processes. Measurements of woody tissue respiration using techniques for measuring oxygen absorption were not feasible at our remote study site, nor on the number of trees presented here. However, given the number of trees sampled, the limited evidence of diurnal variation in CO_2_stem_, and the good replication of tree genera and tree sizes between the plots, we believe that our study does give as accurate a representation as is currently possible of the changes in stem CO_2_ efflux and the proportions of associated *R*_m_ and *R*_g_ which occur as a result of long‐term drought.

Our results suggest that under prolonged periods of drought stress, increasing CO_2_stem_, particularly from small and medium trees, is likely to augment carbon losses from vegetation to atmosphere, which are already likely from drought‐induced mortality. At large scales this response will either further weaken or potentially reverse the tropical forest carbon sink. However, we demonstrate that scaling CO_2_stem_ values to the stand‐scale is currently subject to very high levels of uncertainty, limiting predictions of both the absolute values of stand‐scale CO_2_stem_ and their proportional variation. This will be especially relevant when ecosystems are subject to climatic stresses, such as drought, which are likely to alter ecosystem size structure and related growth, and related physiological‐response regimens.

## Author contributions

The research was designed by L.R., P.M., M.M., A.C.L.d.C., R.S.O., L.V.F. and S.S.V.; data collection, interpretation and analysis was carried out by L.R., A.C.L.d.C., A.A.R.O., P.L.B., P.B.C., A.L.G., A.I.S., I.C., J.L.G., J.A.S.J., M.M. and P.M.; and the manuscript was written by L.R. with contributions from all other authors.

## Supporting information

Please note: Wiley Blackwell are not responsible for the content or functionality of any Supporting Information supplied by the authors. Any queries (other than missing material) should be directed to the *New Phytologist* Central Office.

**Fig. S1** Diurnal variation of stem CO_2_ efflux from trees on the control and TFE plots.
**Fig. S2** Relationships between sapwood depth and tree diameter and basal area.
**Table S1** List of the tree diameter and species of all trees sampled in this study
**Table S2** Distribution of trees across size classes for all trees >10 cm diameter at 1.3 m above ground on the control and TFE plots
**Methods S1** Additional methods relating to the measurement and scaling of stem CO_2_ efflux data.
