# Supplementary material for: Drought stress and tree size determine stem CO 2 efflux in a tropical forest
Source: New Phytol. 2018 Feb 3;218(4):1393–405. doi: 10.1111/nph.15024 (PMC5969101; doi:10.1111/nph.15024)
Supplement: Supplementary file 1 — Fig. S1 Diurnal variation of stem CO2 efflux from trees on the control and TFE plots. Fig. S2 Relationships between sapwood depth and tree diameter and basal area. Table S1 List of the tree diameter and species of all trees sampled in this study Table S2 Distribution of trees across size classes for all trees >10 cm diameter at 1.3 m above ground on the control and TFE plots Methods S1 Additional methods relating to the measurement and scaling of stem CO2 efflux data. [file NPH-218-1393-s001.pdf]

Supplementary Information for *Drought and tree size determine total stem $CO_2$ efflux in a tropical forest*

Lucy Rowland[1*], Antonio C. L. da Costa[2], Alex A. R. Oliveira[3], Rafael S. Oliveira[4], Paulo L. Bittencourt[4], Patricia B. Costa[4], Andre L. Giles[4], Azul I. Sosa[4], Ingrid Coughlin[5], John Godley[10], Steel S. Vasconcelos[7], João A. S. Junior[2], Leandro V. Ferreira[3], Maurizio Mencuccini[8,9], Patrick Meir [6,10]

Methods S1

*Diurnal measurements of* $CO_{2\_stem}$

During October 2013, 20 trees of the dominant genera (Trees shown in Table 1 in Rowland et al 2015) across both plots were fitted with automated open path stem respiration system. A large or small transparent acrylic chamber (large: volume (with foam and tubing) = 676 $cm^3$, surface area =126 $cm^2$; small: volume (with foam and tubing) = 420 $cm^3$, surface area = 90 $cm^2$) was attached to each tree, using a system of straps, as described in the methods section. The chamber was connected to a large storage box which acted as a 145 L buffer volume to minimise fluctuations in $CO_2$ concentration in the delivery air. Air was pumped from the buffer volume to the chamber and from the chamber using two 1.8 L $min^{-1}$ micro diaphragm air pumps, these pumps were connected to manual flow regulators to regulate the flow to between 1-1.3 L $min^{-1}$. A CIRAS1 IRGA (PP Systems, Hitchen, UK), was then used in an open path system to sample the coming from the buffer and going into the chamber (reference), and the air coming out of the chamber (sample, Hutchinson & Livingston 1993). The IRGA was connected to a CR1000 data logger (Campbell Scientific, Logan, USA) which recorded data from the reference and the sample tubes every 15 seconds, alongside air temperature and atmospheric pressure. Respiration was calculated as the difference in $CO_2$ between the reference and the sample lines (moles) multiplied by the moles of gas through the chamber per second, calculated using temperature, pressure and the universal gas constant. The respiration flux was then divided by chamber surface area and converted to $\mu mol\ m^{-2}\ s^{-1}$. The CR1000 also monitored wood temperature from a type T thermocouple inserted into the sapwood of each tree. Two respiration systems were created and left on each tree for a 24 hour period. After installation each system was tested for leaks following the same method listed above. All data was averaged into hourly intervals for each plot. Hours with data for <16 trees were removed, these hours tended to be between 0900 hrs and 1300 hrs when the chambers were changed

over. Due to problems with high humidity during wet season, we were unable to successfully use this equipment in the wet season, hence our estimates are based off dry season only measurements. However during the dry season we found limited diurnal variation in $CO_{2\_stem}$. On the control plot we found a slight decline (mean -3.5±10.7%) in the measurements between 9 am and 7 pm (Fig. S1a). However on the TFE plot we found very limited diurnal variation in $CO_{2\_stem}$ (Fig. S1b), suggesting there was very limited influence of time of day on our measurements during the dry season. Due to very high humidity and water in equipment, data quality was not high enough to use the wet season data, so we assume the same diurnal stability during wet and dry season.

*Measurements of sapwood depth*

In October 2013 tree cores between 15-20 cm in length were extracted at 1.3 m from 21 trees on the TFE and control plot (trees shown in Table 1 in Rowland et al 2015), using an increment borer. In 18 of these tree cores there was a visible change in colour between the sapwood and the heartwood. The sapwood depth in these trees were measured and the (linear) relationships between sapwood depth and diameter, and sapwood area and basal area were plotted (see Fig. S2).

*Scaling* $CO_{2\_stem}$

Method 1: Scaling by surface area

The average wet and dry season $CO_{2\_stem}$ values were multiplied by the total stem surface area per plot, calculated using the following equation S1 from Chambers et al., 2004.

$$SA = 10^\wedge(-0.105 - 0.686 * log10(dbh) + 2.208 * log10(dbh)^2 - 0.627 * log10(dbh)^3) \quad \textit{Equations S1}$$

Where SA is surface area in $m^2$ and dbh is diameter at breast height (1.3 m). These scaling equations are based on highly simplified tree forms, and may not accurately represent the diversity of branching structures which exists in tropical forests.

Method 2: Scaling by sapwood volume

Using a relationship between sapwood depth and diameter (Fig. S1, see above for methods) sapwood area was calculated using basal area, calculated from dbh measured on our sample trees in December 2015. Sapwood area was used to convert $CO_{2\_stem}$ to µmol $m^{-3}$ $s^{-1}$. Using the same basal area-sapwood area relationship applied to all trees we calculated that sapwood area comprised on average 34% of basal area at breast height (1.3 m). Firstly, we assumed that this was fixed across the whole tree volume, which was calculated using the tropical moist forest equations from Chave et al. (2005). The small diameter parts of the tree, particularly branches, are however, likely to comprise greater amounts of sapwood and therefore 34% is likely to be a substantial underestimate. Given that there are no estimates of the proportion of total tree volume that is sapwood within tropical trees, we tested how sensitive our flux values were to an increase of sapwood volume to 50% and 80% of total volume.

Method 3: Scaling by surface area and volume

Using a stem tapering function for tropical trees taken from Chave et al. (2005) we estimated the height of the bole at which 10 cm occurred and calculated the volume of this cylinder between this point and the ground using the dbh of each tree measured in December 2015. The sapwood volume was then calculated by applying the same tapering function to our calculated sapwood area for each tree. The respiration by volume for the wood with >10 cm diameter was calculated and summed and the area of this wood subtracted from the total surface area calculated for each plot (see Method 1). Respiration for the remaining surface area was then calculated following Method 1 and added to the respiration by volume to give plot totals.

$CO_{2\_stem}$ *time-series.*

From October 2013 to February 2016 $CO_{2\_stem}$ measurements on 21 intensively studied trees, of the six most common genera on the control plot and the TFE (trees shown in Table 1 in Rowland et al 2015) were taken using the same method described in the Methods section of this manuscript (closed loop gas analysis measurement method). Measurements were made on the 17/10/2013, 26/10/2013, 08/03/2014, 12/06/2014, 17/06/2014, 10/01/2016, and 10/02/2016. The average values of $CO_{2\_stem}$ from these previous measurements appear higher than we estimate here (Fig. 1b), and this may be because of a limited sample size (only 21 trees per plot). However we use these data to support our finding that differences in $CO_{2\_stem}$ between the control and the TFE plots are consistently elevated with respect to the control plot during the wet season, and more equal during the dry season (Fig. 1b).

References

Chambers, J.Q., Tribuzy, E.S., Toledo, L.C., Crispim, B.F., Higuchi, N., dos Santos, J., Araujo, A.C., Kruijt, B., Nobre, A.D., Trumbore, S.E. (2004) Respiration from a tropical forest ecosystem: Partitioning of sources and low carbon use efficiency. *Ecological Applications* 14, S72-S88.

Chave, J., Andalo, C., Brown, S., Cairns, M. A., Chambers, J. Q., Eamus, D., Folster, H., Fromard, F., Higuchi, N., Kira, T. et al. (2005) Tree allometry and improved estimation of carbon stocks and balance in tropical forests. *Oecologia*, 145, 87-99.

Hutchinson G.L. & Livingston G.P. (1993) Use of chamber systems to measure trace gas fluxes. In *Agricultural Ecosystem Effects on Trace Gases and Global Climate Change,* L. A. Harper, A. R. Mosier, J. M. Duxbury & D. E. Rolston Eds, ASA Spec. Publ. 55. ASA, CSSA, and SSSA, Madison, WI, USA. p. 63-78

Rowland, L., Lobo-do-Vale, R.L., Christoffersen, B.O., Melem, E.A., Kruijt, B., Vasconcelos, S.S., Domingues, T., Binks, O.J., Oliveira, A.A.R., Metcalfe, D., da Costa, A.C.L., Mencuccini, M., Meir, P. (2015) After more than a decade of soil moisture deficit, tropical rainforest trees maintain photosynthetic capacity, despite increased leaf respiration. Global Change Biology 21, 4662-4672.

Supplementary Figures

Figure S1: The percentage variation in the $CO_2$ efflux during a 24 hour period for 20 trees from the control (a.) and TFE (b.) plot during the dry season of 2013 (October). Errors bars indicate the standard error.

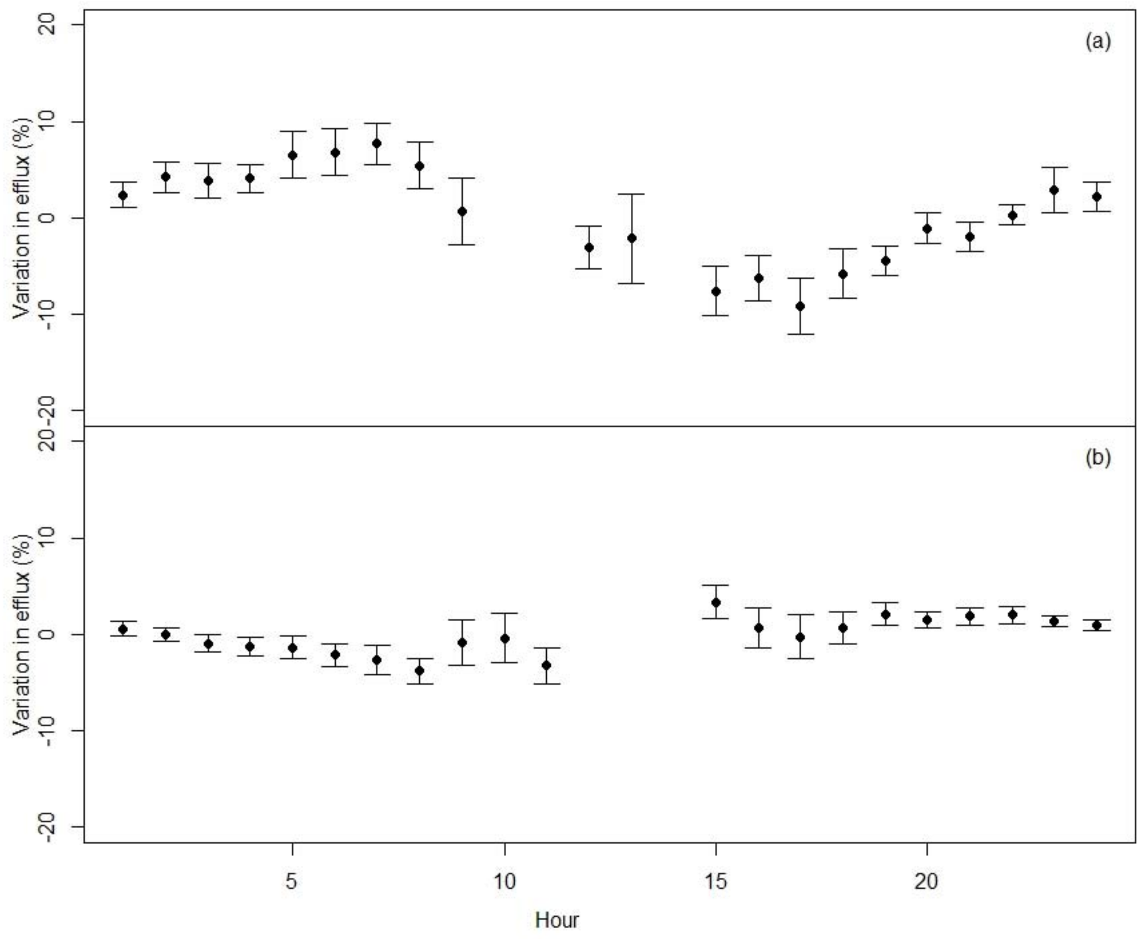

Fig. S2. Relationship between sapwood depth and diameter at breast height (dbh; a.) and the relationship between sapwood area (SA) and basal area (BA; b.), used for scaling by sapwood volume.

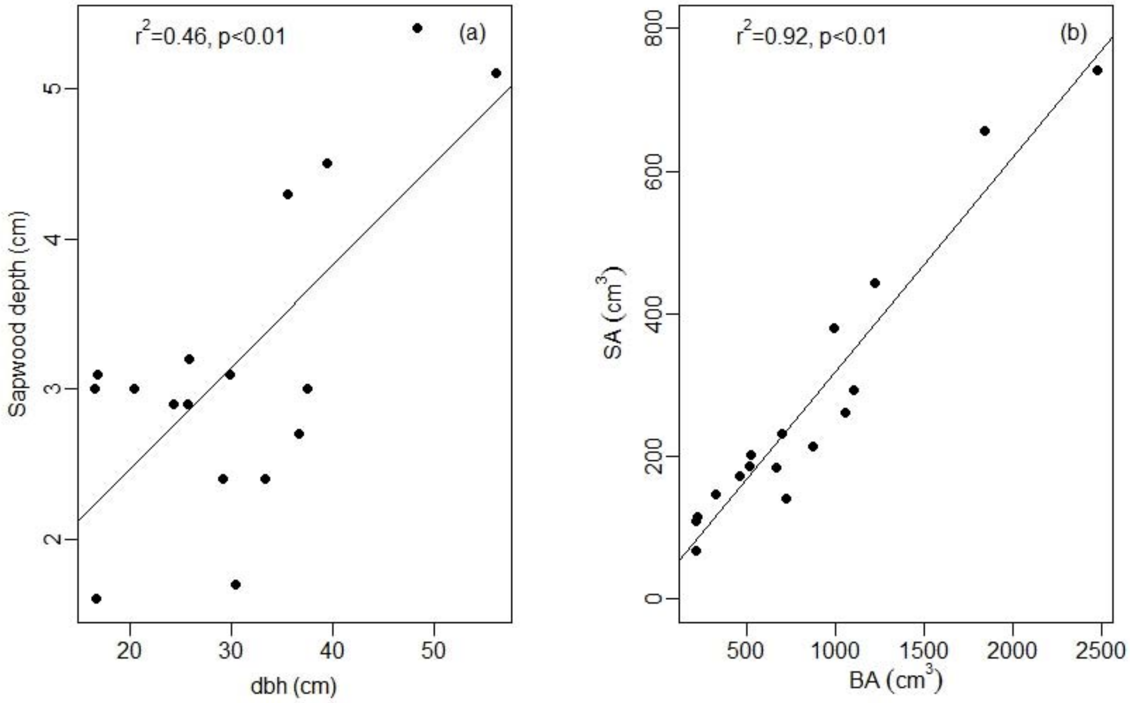

Table S1: List of each tree species sampled, the diameter at breat height of each tree (DBH, cm) and the date a successful stem $CO_2$ efflux measurement (without leaks, see Materials and Methods) were taken from the trees.

| Tr | Pl | Genus | Species | DB | Oc | Oc | Ma | Ju | Ju | Ja | Fe | Oc | Ap |
|---|---|---|---|---|---|---|---|---|---|---|---|---|---|
| 1 | C | Vouacapoua | americana | 20. | | | | | | | | x | x |
| 2 | C | Stryphnode | | 68. | | | | | | | | x | x |
| 3 | C | Vouacapoua | americana | 38. | | | | | | | | x | x |
| 4 | C | Inga | alba | 39. | | | | | | | | x | x |
| 5 | C | Aspidosper | desmanthu | 14. | | | | | | | | x | x |
| 6 | C | Pouteria | anomala | 16. | | | | | | | | x | x |
| 7 | C | Protium | tenuifoliu | 37. | | | | | | | | x | x |
| 8 | C | Qualea | paraensis | 107 | | | | | | | | x | x |
| 9 | C | Swartzia | racemosa | 32. | | | | | | | | x | |
| 10 | C | Vantanea | parvifolia | 47. | | | | | | | | x | x |
| 11 | C | Licania | octandra | 11. | | | | | | | | x | |
| 12 | C | Pouteria | decortican | 24. | | | | | | | | x | x |
| 13 | C | Aspidosper | | 14. | | | | | | | | x | x |
| 14 | C | Eschweilera | grandiflor | 16. | | | | | | | | x | x |
| 15 | C | Aspidosper | nitidum | 33. | | | | | | | | | x |
| 16 | C | Eschweilera | coriacea | 19. | | | | | | | | x | x |
| 17 | C | Vouacapoua | americana | 25. | | | | | | | | x | x |
| 18 | C | Pouteria | gongriijpii | 53. | | | | | | | | x | x |
| 19 | C | Licania | octandra | 18. | | | | | | | | x | x |
| 20 | C | Eschweilera | grandiflor | 18. | | | | | | | | x | x |
| 21 | C | Vouacapoua | americana | 12. | | | | | | | | x | x |
| 22 | C | Pouteria | decortican | 11. | | | | | | | | x | x |
| 23 | C | Pouteria | decortican | 17. | | | | | | | | x | x |
| 24 | C | Pouteria | anomala | 17. | | | | | | | | x | x |
| 25 | C | Minquartia | guianensis | 14. | | | | | | | | x | x |
| 26 | C | Swartzia | racemosa | 21. | | | | | | | | x | x |
| 27 | C | Syzygiopsis | oppositifol | 50. | | | | | | | | | x |
| 28 | C | Vouacapoua | americana | 56. | | | | | | | | x | x |
| 29 | C | Pouteria | guianensis | 51. | | | | | | | | x | x |
| 30 | C | Micropholis | venulosa | 21. | | | | | | | | x | x |
| 31 | C | Pouteria | cladantha | 55. | | | | | | | | x | x |
| 32 | C | Vouacapoua | amaricana | 34. | | | | | | | | x | x |
| 33 | C | Goupia | glabra | 68. | | | | | | | | x | x |
| 34 | C | Vouacapoua | americana | 28. | | | | | | | | x | x |
| 35 | C | Minquartia | guianensis | 15. | | | | | | | | x | x |
| 36 | C | Pouteria | decortican | 20. | | | | | | | | x | x |
| 37 | C | Protium | tenuifoliu | 11. | | | | | | | | x | x |
| 38 | C | Virola | micheli | 47. | | | | | | | | | x |
| 39 | C | Eschweilera | coriacea | 38. | | | | | | | | x | x |
| 40 | C | Micropholis | venulosa | 26. | | | | | | | | x | x |
| 41 | C | Pouteria | anomala | 13. | | | | | | | | x | x |
| 42 | C | Swartzia | racemosa | 47. | | | | | | | | x | x |
| 43 | C | Chrysophyll | manoense | 56. | | | | | | | | x | x |
| 44 | C | Newtonia | suaveolen | 76. | | | | | | | | x | x |
| 45 | C | Inga | gracilifoli | 15. | | | | | | | | x | x |
| 46 | C | Licania | octandra | 23. | | | | | | | | x | x |
| 47 | C | Swartzia | racemosa | 67. | | | | | | | | x | x |

| | | | | | | | | | | | | | |
|---|---|---|---|---|---|---|---|---|---|---|---|---|---|
| 48 | C | Vouacapoua | americana | 21. | | | | | | | | x | x |
| 49 | C | Vouacapoua | americana | 24. | | | | | | | | x | x |
| 50 | C | Pouteria | decortican | 12. | | | | | | | | x | x |
| 51 | C | Licania | membrana | 23. | | | | | | | | x | x |
| 52 | C | Swartzia | racemosa | 26. | | | | | | | | x | x |
| 53 | C | Pouteria | trilocularis | 60. | | | | | | | | x | x |
| 54 | C | Brosimum | potabile | 65. | | | | | | | | x | |
| 55 | C | Inga | alba | 44. | | | | | | | | x | x |
| 56 | C | Micropholis | venulosa | 35. | | | | | | | | x | x |
| 57 | C | Aspidosper | araracanga | 23. | | | | | | | | x | x |
| 58 | C | Pouteria | anomala | 59. | | | | | | | | x | x |
| 59 | C | Goupia | glabra | 92. | | | | | | | | x | |
| 60 | C | Syzygiopsis | oppositifol | 13. | | | | | | | | x | x |
| 61 | C | Sclerolobiu | paraensis | 51. | | | | | | | | x | x |
| 62 | C | Swartzia | racemosa | 29. | | | | | | | | x | x |
| 63 | C | Syzygiopsis | oppositifol | 51. | | | | | | | | x | x |
| 64 | C | Protium | tenuifoliu | 27. | | | | | | | | x | x |
| 65 | C | Pouteria | venulosa | 15. | | | | | | | | x | x |
| 66 | C | Eschweilera | grandiflor | 23. | | | | | | | | x | x |
| 67 | C | Micropholis | venulosa | 62. | | | | | | | | x | x |
| 68 | C | Syzygiopsis | oppositifol | 19. | | | | | | | | x | x |
| 69 | C | Minquartia | guianensis | 12. | | | | | | | | | |
| 70 | C | Pouteria | anomala | 37. | | | | | | | | x | x |
| 71 | C | Minquartia | guianensis | 12. | | | | | | | | x | x |
| 72 | C | Prieurella | | 48. | | | | | | | | x | |
| 73 | C | Pouteria | decortican | 13. | | | | | | | | | x |
| 74 | C | Protium | paniculatu | 13. | | | | | | | | x | x |
| 75 | C | Minquartia | guianensis | 40. | | | | | | | | x | x |
| 76 | C | Swartzia | racemosa | 58. | | | | | | | | x | x |
| 77 | C | Syzygiopsis | oppositifol | 29. | | | | | | | | x | x |
| 78 | C | Syzygiopsis | oppositifol | 34. | | | | | | | | | x |
| 79 | C | Virola | micheli | 27. | | | | | | | | x | x |
| 80 | C | Parkia | ulei | 89. | | | | | | | | x | x |
| 81 | C | Micropholis | venulosa | 18. | | | | | | | | x | x |
| 82 | C | Licania | octandra | 10. | | | | | | | | x | |
| 83 | C | Licania | | 79. | | | | | | | | x | x |
| 84 | C | Protium | tenuifoliu | 19. | | | | | | | | x | x |
| 85 | C | Swartzia | | 73. | | | | | | | | x | x |
| 86 | C | Swartzia | racemosa | 39. | | | | | | | | x | x |
| 87 | C | Vatairea | erythocarp | 63. | | | | | | | | x | x |
| 88 | C | Inga | indet | 18. | | | | | | | | x | x |
| 89 | C | Minquartia | guianensis | 30. | | | | | | | | x | x |
| 90 | C | Syzygiopsis | oppositifol | 64. | | | | | | | | | x |
| 91 | C | Inga | indet | 19. | | | | | | | | x | x |
| 92 | C | Inga | gracilifoli | 18. | | | | | | | | x | x |
| 93 | C | Inga | capitata | 29. | | | | | | | | x | x |
| 94 | C | Eschweilera | grandiflor | 41. | | | | | | | | x | x |
| 95 | C | Inga | rubiginosa | 26. | | | | | | | | x | x |
| 96 | C | Minquartia | guianensis | 16. | | | | | | | | | |

| | | | | | | | | | | | | | |
|---|---|---|---|---|---|---|---|---|---|---|---|---|---|
| 97 | C | Syzygiopsis | oppositifol | 31. | | | | | | | | x | x |
| 98 | C | Licania | membrana | 27. | | | | | | | | x | x |
| 99 | C | Micropholis | venulosa | 29. | | | | | | | | x | x |
| 10 | C | Licania | membrana | 20. | | | | | | | | x | x |
| 10 | C | Eschweilera | grandiflor | 32. | | | | | | | | x | x |
| 10 | C | Minquartia | guianensis | 41. | | | | | | | | x | x |
| 10 | C | Protium | pilossium | 14. | | | | | | | | x | x |
| 10 | C | Prieurella | prieurii | 53. | | | | | | | | x | x |
| 10 | C | Licania | octandra | 21. | | | | | | | | x | x |
| 10 | TF | Vouacapoua | americana | 35. | | 97 | 97 | | | | | x | x |
| 10 | TF | Manilkara | bidentata | 53. | | 10 | 99 | | | | | x | |
| 10 | TF | Guatteria | poeppigia | 15. | | | | | | | | x | x |
| 10 | TF | Vouacapoua | americana | 16. | | | | | | | | x | x |
| 11 | TF | Parkia | N/A | 47. | | | | | | | | x | x |
| 11 | TF | Guatteria | poeppigia | 27. | | | | | | | | x | x |
| 11 | TF | Protium | tenuifoliu | 14. | | | | | | | | x | x |
| 11 | TF | Minquartia | guianensis | 12. | | | | | | | | x | |
| 11 | TF | Guatteria | poeppigia | 22. | | | | | | | | x | x |
| 11 | TF | Dipteryx | Dipteryx | 52. | | | | | | | | x | x |
| 11 | TF | Minquartia | guianensis | 28. | | | | | | | | x | x |
| 11 | TF | Virola | michelii | 34. | | | | | | | | x | x |
| 11 | TF | Protium | tenuifoliu | 33. | | | | | | | | x | x |
| 11 | TF | Syzygiopsis | oppositifol | 59. | | | | | | | | x | x |
| 12 | TF | Mouriri | duckeana | 72. | | | | | | | | x | x |
| 12 | TF | Protium | tenuifoliu | 26. | | | | | | | | x | x |
| 12 | TF | Pouteria | jariensis | 42. | | | | | | | | x | x |
| 12 | TF | Aspidosper | araracanga | 32. | | | | | | | | x | x |
| 12 | TF | Micropholis | venulosa | 15. | | | | | | | | x | x |
| 12 | TF | Vouacapoua | americana | 57. | | | | | | | | x | x |
| 12 | TF | Virola | michelii | 28. | | | | | | | | x | x |
| 12 | TF | Inga | alba | 48. | | | | | | | | x | x |
| 12 | TF | Licania | octandra | 14. | | | | | | | | x | x |
| 12 | TF | Vouacapoua | americana | 45. | | | | | | | | x | x |
| 13 | TF | Minquartia | guianensis | 31. | | | | | | | | x | x |
| 13 | TF | Pouteria | anomala | 25. | | | | | | | | x | x |
| 13 | TF | Pouteria | decortican | 12. | | | | | | | | x | x |
| 13 | TF | Swartzia | racemosa | 10. | | | | | | | | x | x |
| 13 | TF | Swartzia | brachyrac | 14. | | | | | | | | x | x |
| 13 | TF | Diplotropis | purpurea | 44. | | | | | | | | x | x |
| 13 | TF | Protium | pilosimum | 18. | | | | | | | | x | x |
| 13 | TF | Aspidosper | desmanthu | 19. | | | | | | | | x | x |
| 13 | TF | Aspidosper | araracanga | 17. | | | | | | | | x | x |
| 13 | TF | Pouteria | indet | 41. | | | | | | | | x | x |
| 14 | TF | Tetragastris | panamensi | 14. | | | | | | | | x | x |
| 14 | TF | Protium | tenuifoliu | 15. | | | | | | | | x | x |
| 14 | TF | Protium | tenuifoliu | 18. | | | | | | | | x | x |
| 14 | TF | Eschweilera | decolorans | 30. | | | | | | | | x | |
| 14 | TF | Swartzia | racemosa | 42. | | | | | | | | x | |
| 14 | TF | Aspidosper | nitidum | 25. | | | | | | | | x | x |

| | | | | | | | | | | | | | |
|---|---|---|---|---|---|---|---|---|---|---|---|---|---|
| 14 | TF | Pouteria | venosa | 17. | | | | | | | | x | x |
| 14 | TF | Protium | tenuifoliu | 36. | | | | | | | | x | x |
| 14 | TF | Inga | gracilifoli | 17. | | | | | | | | x | x |
| 14 | TF | not | not | 108 | | | | | | | | x | |
| 15 | TF | Inga | alba | 47. | | | | | | | | x | x |
| 15 | TF | Licania | octandra | 21. | | | | | | | | x | x |
| 15 | TF | Tetragastris | panamensi | 23. | | | | | | | | x | |
| 15 | TF | Inga | alba | 56. | | | | | | | | x | x |
| 15 | TF | Xylopia | nitida | 45. | | | | | | | | x | x |
| 15 | TF | Guatteria | poeppigia | 17. | | | | | | | | x | x |
| 15 | TF | Protium | tenuifoliu | 29. | | | | | | | | x | x |
| 15 | TF | Inga | gracilifoli | 25. | | | | | | | | x | x |
| 15 | TF | Licania | egleri | 47. | | | | | | | | x | x |
| 15 | TF | Trattinickia | N/A | 159 | | | | | | | | x | x |
| 16 | TF | Quararibea | guianensis | 67. | | | | | | | | x | x |
| 16 | TF | Erisma | uncinatum | 67. | | | | | | | | x | x |
| 16 | TF | Manilkara | bidentata | 53. | | | | | | | | x | x |
| 16 | TF | Hymenaea | courbaril | 47. | | | | | | | | x | x |
| 16 | TF | Pouteria | decortican | 11. | | | | | | | | x | x |
| 16 | TF | Tetragastris | panamensi | 22. | | | | | | | | x | x |
| 16 | TF | Pouteria | anomala | 20. | | | | | | | | x | x |
| 16 | TF | Pouteria | decortican | 11. | | | | | | | | x | x |
| 16 | TF | Tetragastris | panamensi | 14. | | | | | | | | x | x |
| 16 | TF | Licania | membrana | 17. | | | | | | | | x | x |
| 17 | TF | Tetragastris | panamensi | 12. | | | | | | | | x | x |
| 17 | TF | Licania | octandra | 11. | | | | | | | | x | x |
| 17 | TF | Minquartia | guianensis | 37. | | | | | | | | x | x |
| 17 | TF | Tetragastris | panamensi | 16. | | | | | | | | x | x |
| 17 | TF | Eschweilera | grandiflor | 18. | | | | | | | | x | x |
| 17 | TF | Swartzia | racemosa | 22. | | | | | | | | x | x |
| 17 | TF | Inga | indet | 13. | | | | | | | | x | x |
| 17 | TF | Pouteria | venosa | 41. | | | | | | | | x | x |
| 17 | TF | Eschweilera | grandiflor | 13. | | | | | | | | x | x |
| 17 | TF | Aspidosper | araracanga | 15. | | | | | | | | x | x |
| 18 | TF | Pouteria | anomala | 19. | | | | | | | | x | x |
| 18 | TF | Swartzia | racemosa | 49. | | | | | | | | x | x |
| 18 | TF | Tetragastris | panamensi | 13. | | | | | | | | x | x |
| 18 | TF | Micropholis | venulosa | 13. | | | | | | | | x | x |
| 18 | TF | Aspidosper | araracanga | 28. | | | | | | | | x | x |
| 18 | TF | Licania | membrana | 27. | | | | | | | | x | x |
| 18 | TF | Licania | heteromor | 53. | | | | | | | | x | |
| 18 | TF | Eschweilera | coriacea | 20. | | | | | | | | x | |
| 18 | TF | Minquartia | guianensis | 41. | | | | | | | | x | x |
| 18 | TF | Swartzia | brachyrac | 17. | | | | | | | | x | x |
| 19 | TF | Inga | gracilifoli | 22. | | | | | | | | x | x |
| 19 | TF | Minquartia | guianensis | 19. | | | | | | | | x | x |
| 19 | TF | Virola | michelii | 21. | | | | | | | | x | x |
| 19 | TF | Licania | octandra | 12. | | | | | | | | x | x |
| 19 | TF | Pouteria | anomala | 27. | | | | | | | | x | x |

| | | | | | | | | | | | | | |
|---|---|---|---|---|---|---|---|---|---|---|---|---|---|
| 19 | TF | Licania | membrana | 47. | | | | | | | | x | x |
| 19 | TF | Vouacapoua | americana | 33. | | | | | | | | x | x |
| 19 | TF | Inga | gracilifoli | 34. | | | | | | | | x | x |
| 19 | TF | Eschweilera | grandiflor | 13. | | | | | | | | x | x |
| 19 | TF | Vouacapoua | americana | 15. | | | | | | | | x | x |
| 20 | TF | Pouteria | decortican | 10. | | | | | | | | x | x |
| 20 | TF | Pouteria | jariensis | 51. | | | | | | | | x | x |
| 20 | TF | Licania | octandra | 17. | | | | | | | | x | x |
| 20 | TF | Minquartia | guianensis | 25. | | | | | | | | x | x |
| 20 | TF | Syzygiopsis | oppositifol | 63. | | | | | | | | | x |
| 20 | TF | Micropholis | egensis | 23. | | | | | | | | x | x |
| 20 | TF | Couma | macrocarp | 44. | | | | | | | | x | |
| 20 | TF | Swartzia | racemosa | 63. | | | | | | | | x | x |
| 20 | TF | Eschweilera | coriacea | 18. | | | | | | | | x | x |
| 20 | TF | Pouteria | venosa | 10. | | | | | | | | x | x |
| 21 | TF | Protium | paniculatu | 38. | | | | | | | | x | |
| 21 | TF | Vouacapoua | americana | 53. | | | | | | | | x | x |
| 21 | TF | Virola | crebrinerv | 20. | | | | | | | | x | x |
| 21 | TF | Ocotea | rubra | 47. | | | | | | | | x | x |
| 21 | TF | Not | Not | | | | | | | | | | x |
| 21 | TF | Minquartia | guianensis | 16. | | | | | | | | x | |
| 21 | C | Manilkara | bidentata | 32. | x | x | x | x | x | x | x | | |
| 21 | C | Pouteria | anomala | 15. | x | x | x | x | x | x | | | |
| 21 | C | Pouteria | anomala | 16. | x | x | x | x | x | x | x | | |
| 21 | C | Protium | ifolium | 29. | x | x | x | x | x | x | | | |
| 22 | C | Manilkara | bidentata | 32. | x | x | x | x | x | x | x | | |
| 22 | C | Swartzia | racemosa | 30. | x | x | x | x | x | x | x | | |
| 22 | C | Eschweilera | grandiflor | 34. | x | x | x | x | x | x | x | | |
| 22 | C | Protium | ifolium | 24. | x | x | x | x | x | x | | | |
| 22 | C | Eschweilera | ra coriacea | 37. | x | x | x | x | x | x | x | | |
| 22 | C | Eschweilera | a coriacea | 24. | x | x | x | x | x | | x | | |
| 22 | C | Licania | ranacea | 20. | x | x | x | x | x | x | x | | |
| 22 | C | Swartzia | racemosa | 45. | x | x | | x | x | | x | | |
| 22 | C | Licania | ranacea | 23. | x | x | x | x | x | x | x | | |
| 22 | C | Eschweilera | grandiflor | 35. | x | x | x | x | x | x | x | | |
| 23 | C | Manilkara | bidentata | 48. | x | x | | x | x | x | x | | |
| 23 | C | Pouteria | anomala | 35. | x | x | x | x | x | x | x | | |
| 23 | C | Protium | ifolium | 33. | x | x | x | x | x | x | x | | |
| 23 | C | Swartzia | racemosa | 37. | x | x | x | x | x | x | x | | |
| 23 | TF | | anomala | 25. | x | x | x | | x | x | x | | |
| 23 | TF | Manilkara | bidentata | 56. | x | x | x | x | x | x | x | | |
| 23 | TF | Protium | ifolium | 29. | x | x | x | x | x | x | x | | |
| 23 | TF | Swartzia | racemosa | 40. | x | x | x | x | x | x | x | | |
| 23 | TF | Eschweilera | ra coriacea | 36. | x | x | x | x | x | x | x | | |
| 23 | TF | Eschweilera | grandiflor | 24. | x | x | x | x | x | x | x | | |
| 24 | TF | Protium | ifolium | 27. | x | x | x | x | x | x | x | | |
| 24 | TF | Pouteria | anomala | 20. | x | x | x | x | x | x | x | | |
| 24 | TF | Licania | ranacea | 15. | x | x | x | x | x | x | x | | |
| 24 | TF | Manilkara | bidentata | 36. | x | x | x | x | x | x | x | | |

| | | | | | | | | | | | | | |
|---|---|---|---|---|---|---|---|---|---|---|---|---|---|
| 24 | TF | Eschweilera | grandiflor | 16. | x | x | | x | x | x | x | | |
| 24 | TF | Swartzia | racemosa | 21. | x | x | x | x | x | x | x | | |
| 24 | TF | Licania | ranacea | 25. | x | x | x | x | x | x | x | | |
| 24 | TF | Eschweilera | ra coriacea | 32. | x | x | x | x | x | x | x | | |
| 24 | TF | Swartzia | racemosa | 36. | x | x | x | x | x | | x | | |
| 24 | TF | Manilkara | bidentata | 29. | x | x | x | x | x | x | x | | |
| 25 | TF | Eschweilera | ra coriacea | 26. | x | x | x | x | x | x | x | | |
| 25 | TF | Pouteria | anomala | 27. | x | x | x | x | x | x | x | | |
| 25 | TF | Licania | ranacea | 47. | x | x | x | x | x | x | x | | |
| 25 | TF | Eschweilera | grandiflor | 16. | x | x | x | x | x | x | x | | |

Table S2: Distribution of all trees on the Control and TFE used for the scaling exercise in Table 2, into the size categories of diameter at breast height (cm) used in Figure 2

| | <15 | 15-20 | 20-25 | 25-30 | 30-40 | 40-50 | 50-60 | >60 |
|---|---|---|---|---|---|---|---|---|
| Control | 158 | 93 | 71 | 45 | 55 | 21 | 14 | 24 |
| TFE | 137 | 97 | 52 | 43 | 45 | 25 | 13 | 8 |
